# Effect of epoch length on intensity classification and on accuracy of measurement under controlled conditions on treadmill: Towards a better understanding of accelerometer measurement

**DOI:** 10.1371/journal.pone.0227740

**Published:** 2020-01-24

**Authors:** Nicolas Fabre, Léna Lhuisset, Caroline Bernal, Julien Bois

**Affiliations:** Universite de Pau & des Pays de l’Adour, e2s UPPA, MEPS, Tarbes, France; University of Bourgogne France Comté, FRANCE

## Abstract

**Purpose:**

The aim of this study was to analyze the effect of epoch length on intensity classification during continuous and intermittent activities.

**Methods:**

Ten active students exercised under controlled conditions on a treadmill for four 5-min bouts by combining two effort intensities (running and walking) and two physical activity (PA) patterns (continuous or intermittent). The testing session was designed to generate a known level of moderate to vigorous PA (MVPA) for each condition. These PA levels were used as criterion measures to compare with the accelerometer measures. Data obtained from the accelerometer were reintegrated into 1-sec, 10-sec, 30-sec and 60-sec epochs. Equivalence testing was used to examine measurement agreements between MVPA values obtained with the different epochs and the reference values. Mean absolute percent errors (MAPE) were also calculated to provide an indicator of overall measurement error.

**Results:**

During the intermittent conditions, only the value obtained with the 1-sec epoch was significantly equivalent to the reference value. With longer epochs the difference increased for both intermittent conditions but in an opposite way: with longer epochs, MVPA decreased during walking but increased during running. Regarding the measurement accuracy, the pattern of variations according to the epoch length selected during the intermittent conditions was identical between walking and running: MAPE increased with the increase in epoch length. MAPE remained low only for the 1-sec epoch (7.6% and 2.7% for walking and running, respectively), increased at 31.3% and 34% for the 10-sec epoch and until near 100% with the 30- and 60-sec epoch lengths.

**Conclusion:**

This study highlighted the misclassification of exercise intensity based on accelerometer measurement and described for the first time the extent and the direction of this misclassification. Moreover, we can confirm that the shorter epochs are more accurate to measure the real exercise intensity during intermittent PA whatever the intensity.

## Introduction

Accelerometers are useful tools to assess objectively physical activity (PA) level in a wide range of population (*i*.*e*., children and adolescents [1], adults [2] or elderly [3]). Several studies evaluated their validity to assess PA intensity compared to indirect calorimetry or doubly labeled water in adults [4–8] or in children [9]. All these studies usually revealed that accelerometers were more accurate to estimate energy expenditure in a laboratory context than in a real life context. One of the reasons for this difference can be found in the intermittent nature of the activities encountered in a real life context. The validity of the accelerometers is therefore questionable for specific intermittent activities such as those observed during many field and court sports or during free-play and unstructured games like practiced by many children during recess [10] or during whole day measurement spontaneous and intermittent by nature especially for children and adolescent [11]. Specifically, the moderate-to-vigorous PA (MVPA) duration, an essential variable in terms of health used as a basis of the guidelines for the world public health authorities, appears to be strongly affected during this kind of activity pattern.

The problem with the MVPA measurement during intermittent activities comes mainly from the sampling interval, also called epoch length—an epoch representing the length of time during which activity counts (a measure of activity magnitude) are summed before being stored by the accelerometer -. Indeed, it is now well established that the selection of different epoch lengths during data processing leads to different MVPA scores [12–17]. Among these studies, some observed a decrease in MVPA scores as the epoch lengths increase [13–15] while others found the opposite with an increase in MVPA with longer epoch lengths [16, 17]. It is therefore difficult to establish a general rule, especially since a common limitation of all these studies is the lack of measurement criterion, such as direct observation, thus preventing comparison and determination of which epoch length is actually the most accurate to estimate PA. However, it seems that the PA context may have some influence on the effects of epoch length on MVPA estimates. A decrease in MVPA scores as the epoch lengths increase is generally observed in whole-day PA measurement studies [13–15] whereas an increase in MVPA scores is reported during specific and structured activities such as physical education (PE) lessons [16]. Interestingly, Sanders et al. [17] studied both these PA contexts in an unique study highlighting an increase in estimates of MVPA as epoch length increased from 1-sec to 60-sec during PE lessons and, on the contrary, a decreased during a whole-day measurement period.

It seems therefore that the effect of epoch length on the assessment of MVPA may vary depending on the nature and the intensity of the activity. Thus, it is important to assess and clearly understand the direction (*i*.*e*., an increase or a decrease in MVPA estimates) and the extent (*i*.*e*., the measurement accuracy) of any misclassification of MVPA when selecting the epoch length during different activity patterns in terms of intensity (*i*.*e*., walking or running) and modality (*i*.*e*., continuous or intermittent activity).

Thus, the aim of this study was to analyze the effect of epoch lengths on intensity classification during continuous or intermittent activities. We used four controlled conditions on the treadmill by combining two different effort intensities (running and walking) and two different PA patterns (continuous or intermittent). The treadmill speeds were chosen to induce moderate- and vigorous-intensity PA (MVPA) when the participants were respectively walking or running [18]. This way, the accelerometer measurement can be compared to a criterion measure. We hypothesized that in continuous effort conditions, epoch length would not affect MVPA level, regardless of its intensity (*i*.*e*., running or walking). In the intermittent condition, we hypothesized that MVPA level would be affected by epoch length but this effect would vary as a function of intensity: for higher intensity (running) we hypothesized that longer epoch would result in higher MVPA level whereas for moderate intensity (walking) longer epoch length would lead to lower levels of MVPA. Regarding accuracy we hypothesized that shorter epoch would result in greater measurement accuracy.

## Materials and methods

### Participants

Ten active and healthy students in sport sciences (7 males) participated in this study. Their age was 28.4±9.9 years, height 173.3±8.8 cm and body mass 65.7±10.5 kg. They all had previous experience of walking and running on a treadmill. The participants refrained from ingesting caffeine or alcohol for at least 12h before testing, ate a light meal 3h before testing and refrained from strenuous exercise before the testing session. The study protocol complied with the declaration of Helsinki for human experimentation. Possible risks and benefits were explained, and written informed consent was obtained from each subject before participation in line with the procedures set by our University ethics committee for human research. The Institute review board CPP Sud Méditerranée III approved all procedures.

### Experimental design

Since only submaximal intensities were used during the protocol, all the exercise conditions were performed within one day.

After a ten-minute warm-up/familiarization period at a self-selected and preferred speed, participants completed four 5-min bouts of exercise on a motorized treadmill (Zebris Medical GmbH, Germany) in a random order and with a 5-min rest period between: (i) one intermittent exercise involving alternating intervals of 15-sec walk at 5 km.h^-1^ and 15-sec passive recovery; (ii) one intermittent exercise involving alternating intervals of 15-sec run at 10 km.h^-1^ and 15-sec passive recovery (iii) one continue exercise involving walking at 5 km.h^-1^ and (iv) one continue exercise involving running at 10 km.h^-1^. This testing session was designed to generate MVPA when the participant was walking or running on the treadmill since walking at 5 km.h^-1^ is already recognized as absolute moderate and vigorous intensity [18, 19]. During the resting period, participants were instructed to stay in a standing position on the treadmill moving as little as possible.

### Measurement

Participants wore the tri-axial GT3X+ accelerometer (Actigraph, Pensacola, FL, USA) to assess their PA levels during the entire testing session.

The GT3X+ is a small and light-weight device that measures movement in three axes from a triaxial accelerometer (range ± 6 g). The device was secured with an elastic belt on the participant’s right hip.

### Data analysis

The GT3X+ was set to collect data at 30 Hz. Data were downloaded and copies of the original files obtained from the accelerometer were made and then reintegrated into 1-sec, 10-sec, 30-sec and 60-sec epochs resulting in four copies of each record differentiated only by epoch length. The data were processed using the ActiLife software (version 6.13.3, ActiGraph Inc, Pensacola, FL, USA). The GT3X+ was synchronized to the time of the computer clock. During the testing session, the starting time of each exercise bout was read on the computer used to set the GT3X+ and annotated. We then analyzed each exercise bout separately using the time filtering function of the ActiLife software.

Troiano cut-points [20, 21] were applied to vertical axis data in order to calculate time spent in different physical activity (PA) intensities. More specifically, cut-off points (based on the 60-sec epoch) were: <99 counts/min for sedentary time (SED), >100 and <2019 counts/min for light physical activity (LPA), >2020 and <5998 counts/min for moderate physical activity (MPA) and >5999 counts/min for vigorous physical activity (VPA). Cut-off points values were then divided for data reintegrated into 1-sec, 10-sec and 30-sec epoch lengths and consequently used to calculate time spent in the different intensities. Time spent in MVPA was determined by adding time in MPA and VPA.

### Statistical analysis

All results are presented as means ± standard deviations (SD).

We used equivalence testing to statistically examine measurement agreements between estimated duration of MVPA values obtained with the different epoch lengths selected and the reference values. In traditional hypothesis testing, the focus is on testing for a significant difference. Failing to reject the null hypothesis (e.g., that two methods are not different) allows one to conclude that there is no evidence of a difference. However, this does not necessarily imply that the estimates are equivalent [22]. Using an equivalence test, allows one to determine whether a method is ‘‘significantly equivalent” to a reference. With these types of analyzes, it is important to specify an appropriate equivalence zone before testing. We selected a 10% error zone, which represented ± 30-sec for the continuous conditions and ± 15-sec for the intermittent conditions, as proposed by Kim et al. [23] who used the same procedure to examine measurement agreement between the accelerometer and a criterion measure (*i*.*e*., a gas analyzer). With a 95% equivalence test (*i*.*e*., an alpha of 5%), an estimate is considered to be equivalent to the reference value (with 95% precision) if the 90% confidence interval (CI) for a mean of the estimated measurement falls into the proposed equivalence zone (*i*.*e*., 10% of the mean) of the reference value.

Mean absolute percent errors (MAPE) were also calculated to provide an indicator of overall measurement error. MAPE were computed as the average of absolute differences between the MVPA values obtained with the different epoch lengths and the reference value (*i*.*e*., MVPA = 5-min for continuous conditions and MVPA = 2.5-min for intermittent conditions) divided by the reference value, multiplied by 100 [24]. This is a more conservative estimate of error that takes into account both overestimation and underestimation because the absolute value of the error is used in the calculation.

A 1-way ANOVA for repeated measurements was chosen to test for differences between epochs in time spent at each intensity on the one hand and MAPE on the other hand, during intermittent conditions. When an overall difference was found, the Scheffe post hoc test was used for specifics comparisons. Statistical significance was accepted at p<0.05 excepted when multiple tests were used across the different conditions. In these cases, Bonferroni adjustments to protect against type I error have been applied. Thus, differences were considered significant when p<0.05/4 = 0.0125. Data were analyzed with the software package Statistica version 12.5.

## Results

### Effect of epoch length on intensity classification

Time spent at the different PA levels according to the different epochs selected is presented in Table 1.

**Table 1:** Number of minutes recorded for each condition and at each intensity according to the epoch length selected.

	**Continuous walking**				**Continuous running**			
**epoch**	**SED**	**LPA**	**MPA**	**VPA**	**SED**	**LPA**	**MPA**	**VPA**
	^**(min)**^	^**(min)**^	^**(min)**^	^**(min)**^	^**(min)**^	^**(min)**^	^**(min)**^	^**(min)**^
**1-sec**	0.09±0.06	0.06±0.04	4.85±0.07	0.00±0.00	0.08±0.06	0.02±0.01	0.02±0.01	4.88±0.07
**10-sec**	0.05±0.08	0.13±0.07	4.82±0.09	0.00±0.00	0.03±0.07	0.00±0.00	0.15±0.05	4.82±0.09
**30-sec**	0.00±0.00	0.15±0.24	4.85±0.24	0.00±0.00	0.00±0.00	0.00±0.00	0.12±0.21	4.88±0.21
**60-sec**	0.00±0.00	0.00±0.00	5.00±0.00	0.00±0.00	0.00±0.00	0.00±0.00	0.00±0.01	4.99±0.01
	**Intermittent walking**				**Intermittent running**			
**epoch**	**SED**	**LPA**	**MPA**	**VPA**	**SED**	**LPA**	**MPA**	**VPA**
	^**(min)**^	^**(min)**^	^**(min)**^	^**(min)**^	^**(min)**^	^**(min)**^	^**(min)**^	^**(min)**^
**1-sec**	2.32±0.08	0.37±0.09	2.31±0.08	0.00±0.00	2.24±0.06	0.20±0.05	0.22±0.04	2.35±0.08
** **	^a^^,^^b^^,^^c^	^a^^,^^b^^,^^c^	^a^^,^^b^^,^^c^		^a^^,^^b^^,^^c^	^a^	^a^^,^^b^^,^^c^	^a^^,^^b^^,^^c^
**10-sec**	0.75±0.71	2.53±0.90	1.72±0.26	0.00±0.00	0.62±0.59	1.03±0.50	1.53±0.70	1.82±0.59
** **		^b^^,^^c^	^b^^,^^c^			^b^^,^^c^	^b^^,^^c^	^b^^,^^c^
**30-sec**	0.00±0.00	4.85±0.87	0.15±0.47	0.00±0.00	0.00±0.00	0.05±0.16	4.75±0.63	0.20±0.63
** **								
**60-sec**	0.00±0.00	4.90±0.32	0.10±0.32	0.00±0.00	0.00±0.00	0.00±0.00	4.70±0.95	0.30±0.95
** **								

SED: sedentary time; LPA: light physical activity time; MPA: moderate physical activity time; VPA: vigorous physical activity time

^a^ Significantly different from the 10-sec epoch with p<0,001

^b^ Significantly different from the 30-sec epoch with p<0,001

^c^ Significantly different from the 60-sec epoch with p<0,001

As hypothesized, during continuous walking or running, more than 95% of the time was spent in MPA (for walking condition) or VPA (for running condition) whatever the epoch selected (see Table 1).

During intermittent walking, as epoch length increased, SED and MPA times decreased (*F*(3,27) = 53.02, p<0.001 and *F*(3,27) = 168.71, p<0.001, respectively) while LPA time increased (*F*(3,27) = 175.87, p<0.001). Thus, with the longest epoch (60-sec) the whole intermittent walking condition (5-min) was classified as LPA whereas only 2.5-min were actually walked.

During intermittent running, as epoch length increased, SED and VPA times decreased (*F*(3,27) = 77.34, p<0.001 and *F*(3,27) = 58.70, p<0.001, respectively) and at the same time, MPA time increased (*F*(3,27) = 233.47, p<0.001). Thus, with the longest epoch (60-sec) the whole intermittent running condition (5-min) was classified as MPA whereas only 2.5-min were actually run.

**Fig 1:**
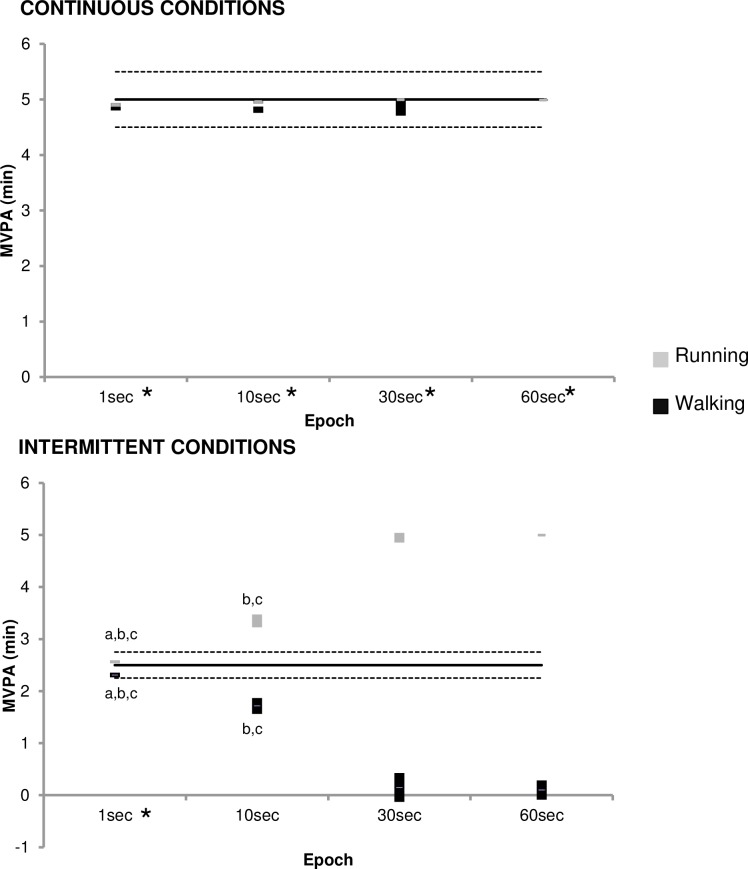
Results from 95% equivalence testing for agreement in time MVPA according to the different epoch lengths selected. Upper and lower panels represent data from the continuous and intermittent conditions, respectively. Dark lines represent the reference values (*i*.*e*., 5-min for continuous conditions and 2.5-min for intermittent conditions) and the dotted lines indicate the equivalence zone (±10% of the reference value); Grey and dark bars represent the 90% confidence interval for the mean MVPA value obtained with the corresponding epoch. * Within the equivalence zone. ^a^ Significantly different from the 10-sec epoch with p<0,001. ^b^ Significantly different from the 30-sec epoch with p<0,001. ^c^ Significantly different from the 60-sec epoch with p<0,001.

Moreover, the use of equivalence testing made it possible to determine whether the MVPA obtained with the various epoch lengths was equivalent to the reference value. The calculated 90% CI for the mean MVPA was compared with the computed equivalence zone for the reference value. This is illustrated in Fig 1. During the continuous conditions, the MVPA was always significantly equivalent to the reference 5-min value. This is shown by the fact that the 90% CI of MVPA obtained with the various epoch lengths were completely within the equivalence zone (lower bound = 4.5min, upper bound = 5.5min). During the intermittent conditions, only the values obtained with the 1-sec epoch were significantly equivalent to the reference 2.5min value. With longer epoch lengths the differences with the reference value increased for both intermittent walking and running conditions but in an opposite way: with longer epoch lengths, MVPA during walking decreased but increased during running (see Fig 1).

To summarize, during intermittent conditions, the use of a 1-sec epoch length leaded to a good classification of SED and MPA for walking or VPA for running and using larger epochs led to different types of misclassification: intermittent walking was misclassified as LPA when it should be classified as MPA whereas intermittent running was misclassified as MPA when it should be classified as VPA.

### Effect of epoch length on measurement accuracy

**Fig 2:**
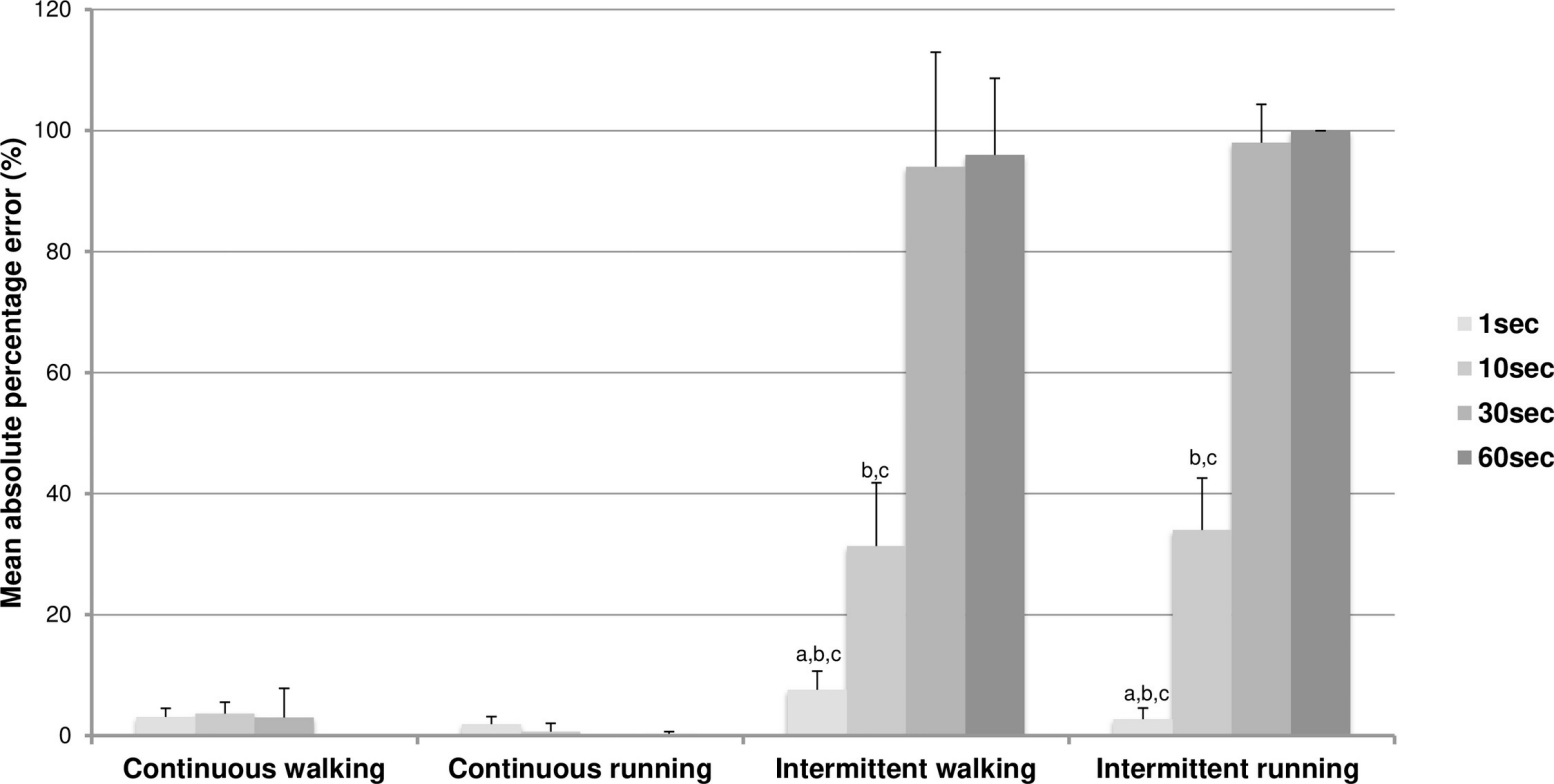
Mean absolute percentage error (±SD) between measurements with the different epochs and the reference value (*i*.*e*., MVPA = 5-min for continuous conditions and MVPA = 2.5-min for intermittent conditions). ^a^ Significantly different from the 10-sec epoch with p<0,001. ^b^ Significantly different from the 30-sec epoch with p<0,001. ^c^ Significantly different from the 60-sec epoch with p<0,001.

Measurement accuracy has been estimated by the calculation of the MAPE for each condition and each epoch. It is represented in Fig 2.

The magnitude of errors was very low to null for the continuous conditions whatever the intensity of the activity (i.e. walking or running) and the epoch length selected (form 0 to 3.7%). During the intermittent conditions, the pattern of variations according to the epoch length selected was identical between walking and running with an increasing MAPE with the increase in epoch length. MAPE remained low only for the 1-sec epoch (7.6% and 2.7% for walking and running, respectively) and then increased at 31.3% and 34% for the 10-sec epoch for walking and running and until near 100% with the 30-sec and 60-sec epochs lengths. During walking, MAPE at 1-sec epoch was statistically different with MAPE at 10-sec, 30-sec and 60-sec epoch (*F*(3,27) = 178.71, p<0.001). MAPE at 10-sec epoch was statistically different with MAPE at 1-sec, 30-sec and 60-sec epoch (*F*(3,27) = 178.71, p<0.001). In the same way during running, MAPE at 1-sec epoch was statistically different with MAPE at 10-sec, 30-sec and 60-sec epoch (*F*(3,27) = 652.11, p<0.001). MAPE at 10-sec epoch was statistically different with MAPE at 1-sec, 30-sec and 60-sec epoch (*F*(3,27) = 652.11, p<0.001). No difference was observed between MAPE at 30- and 60-sec epoch during walking (*F*(3,27) = 178.71, p = 0.98) or during running (*F*(3,27) = 652.11, p = 0.99).

## Discussion

This study analyzed, under controlled conditions (*i*.*e*., laboratory testing on treadmill), the effect of epoch length on intensity classification during continuous or intermittent activities. In this very standardized context (i.e., exercise duration and intensity controlled and similar for all participants), it was possible to obtain a criterion measure of the PA level. To our knowledge, usually, no criterion measure was provided in studies interested in the effect of epoch length on PA classification. Therefore, it was possible to objectively verify the hypothesis of the study and to well understand the extent and the direction of any misclassification. As expected, we observed that during continuous exercise, the PA estimates from the accelerometer were accurate regardless of the epoch length selected. This result was quite obvious but permitted us to classify the PA level obtained when a person is moving at 5 or 10 km.h^-1^: the 5-min walking bout at 5 km.h^-1^ was classified as MPA for almost 100% of the total duration and the 5-min running bout at 10 km.h^-1^ was classified as VPA for almost 100% of the total duration. During intermittent conditions, given that the PA criterion measures for the intermittent conditions were 2.5-min of MPA and 2.5-min of SED for walking and 2.5-min of VPA and 2.5-min of SED for running, since these conditions consisted in alternating intervals of 15-sec exercise and 15-sec passive recovery during a total rime of 5-min, it appeared that, whatever the exercise intensity, only the 1-sec epoch length was accurate to estimate the right MVPA value. Already from the 10-sec epoch length, the estimate of MVPA was out of the equivalent zone and the MAPE was around 30%. The difference was even more important for the 30- and 60-sec epoch lengths with a MAPE around 100%. The direction of these misclassifications varied according to the exercise intensity: for high intensity (*i*.*e*., running condition), the estimate of MVPA increased as epoch length increased. This increase was probably due to the misclassification of SED time as MPA, as indicated by SED decreasing and MPA increasing as epoch length increased. On the contrary, for lower intensity (*i*.*e*., walking condition), the estimate of MVPA decreased as epoch length increased which can be explained by the misclassification of MPA bouts as LPA, as indicated by LPA increasing as epoch length increased. In other words, during our intermittent conditions, exercise intensity was averaged as the epoch length increased.

These findings explain the conflicting results of previous studies about the direction of the MVPA misclassification. Some studies obtained a decrease estimate of MVPA with the increase in epoch length [13–15, 25] and others obtained the opposite [16, 17]. Overall, it appears that in the majority of the studies showing a decrease in the MVPA estimate, the measurements were made in a whole-day measurement context whereas in the studies showing an increase in MVPA, the measurements were made in a PE context. Considering the recommended amount of MVPA during PE lesson (*i*.*e*., 50% of the PE time [26]) *vs* the general daily recommendations (*i*.*e*., 30-min of MVPA for adults and 60-min for children and adolescents [27]), the percentage of high intensity PA during a whole-day measurement context is much lower than during PE. Thus our intermittent walking condition approximated the whole-day measurement context in terms of PA pattern and intensity. By contrast, our intermittent running condition came closer to the context of a PE lesson with higher exercise intensity. We can confirm the hypothesis proposed by Aibar and Chanal [16] or by Sanders et al. [17] who compared the influence of epoch length during PE and whole-day measurement context in adolescents and who suggested that the context where PA occurs influences the effect of epoch length on PA estimates. To be more precise about the context of PA, it is the exercise intensity that will define the direction of the epoch length effect. However, it appears that in any case, the 1-sec epoch gives the better estimate of the amount of MVPA that has been practiced.

There are several limitations that should be considered when interpreting the present findings. On a first sight, the small size sample might temper our conclusions and limit their application to a wider population in comparison to the sample size usually used in previous studies on this topic. However, our extremely controlled trials on treadmill limited the between subjects measurement variability with exactly the same duration of activity and recovery, the same speed for every single subject, giving us confidence in our results. Secondly, the transfer of the findings in an ecological environment is questionable. Obviously, this protocol was very far from a whole-day measurement or a PE context but we believe that the laboratory context was necessary in order to understand clearly the extent and the direction of any misclassification of PA levels during intermittent activities.

## Conclusion

This study documents for the first time, the extent (*i*.*e*, from less than 10% MAPE with a 1-sec epoch length to 30% with a 10-sec epoch length and to 100% with 30- or 60-sec epoch length, whatever the intensity) and the direction (*i*.*e*., a decrease in MVPA estimates for low intensity PA or an increase in MVPA estimates for high intensity PA as epoch length increased) of the misclassification of PA level based on accelerometer measurement. Moreover, we can confirm that the shorter epoch lengths are the more accurate for capturing the real PA levels during intermittent PA whatever the intensity. Therefore it becomes apparent that comparisons between studies using short (<10-sec) or long (>10-sec) epochs should not be made without reference to the present results. Our findings help to understand and analyze different PA levels obtained in the same conditions but with a different epoch lengths.

### Perspectives

During the intermittent conditions, exercise intensity was somehow averaged as the epoch length increased and, as discussed above, accuracy was lost with long epoch lengths in the quantification of actual PA. The exact PA (in terms of exercise intensity and duration) realized by a person, can be regarded as the external load. The internal load can be obtained by the assessment of energy expenditure. During intermittent activities, the external load measurement does not take into consideration the impact of a previous exercise bout following a recovery phase, as does the internal load measurement. Accelerometers often assess daily PA levels from energy expenditure estimates [28]. It appears therefore questionable if shorter epochs are effectively more accurate to estimate energy expenditure during intermittent activities. Thus, it would be interesting to duplicate this protocol with a direct oxygen consumption measurement and use the energy expenditure result obtained as criterion measure.

## Supporting information

S1 Data (XLSX)
